# A simplified methodology for the activation of organic charcoal

**DOI:** 10.1016/j.mex.2023.102241

**Published:** 2023-06-13

**Authors:** Stephen Nelson, Connor Hare, Atieno Mpyisi, Chen Wei Yew, Kendra LeBrun, Christina R. Harris, Berquin D. Feese, Ronald Celestine, Masato Kinjo, John S. Peacock

**Affiliations:** Weimar University, 20601W. Paoli Ln., Weimar, CA 95736, USA

**Keywords:** Biochar, Spectrophotometric analysis, Thermal air oxidation, Water treatment, Activation of charcoal

## Abstract

One in three people globally are challenged to live on hazardous, unsanitary water, and this relates to higher risks of death and development of diseases. According to scientific research, activated charcoal can be used to clean water contaminants to help make water safer.

• Carbonization: this study demonstrates an inexpensive method of producing activated charcoal that can be performed in any setting using locally available biomass materials.
• Activation: thermal air oxidation between moderate temperatures of 450–550 °C.

Our data indicate that this technique produces charcoal with an adsorptive capacity near to that of commercial-grade charcoal as demonstrated by spectrophotometric analysis. This simple approach to charcoal activation may benefit rural communities where sources of sanitary water are low or nonexistent.

## Graphical abstract

**Figure:**
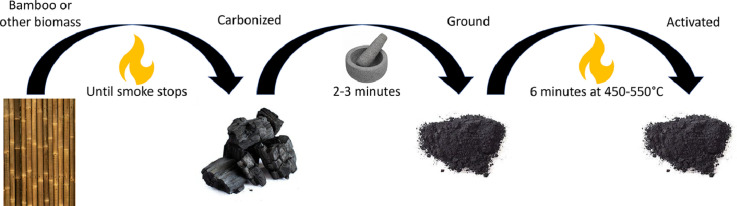

Specifications table

**Table:** 

Subject area:	Environmental Science
More specific subject area:	Environmental chemistry
Name of your method:	Activation of charcoal
Name and reference of original method:	Cobb, A., Warms, M., Maurer, E. P., & Chiesa, S. (2012). Low-Tech Coconut Shell Activated Charcoal Production. International Journal for Service Learning in Engineering, Humanitarian Engineering and Social Entrepreneurship, 7(1), 93–104. 10.24908/ijsle.v7i1.4244
Resource availability:	NA

## Method details

### Materials

Step 1 (Carbonization)

• 6 g. bamboo shavings
• Propane stove
• Metal box (about 15.24x7.62x10.16 cm)
• Metal lid with 6 evenly spaced 1.5 mm holes
• Mortar and pestle

Step 2 (Activation)

• Small metal mint box with lid
• Carbonized bamboo
• Propane stove
• Infrared thermometer gun
• Stainless steel lab spatula
• Timer

Step 3 (Testing)

• 0.20 g. activated charcoal
• 25 mL methyl orange solution
• Laboratory beaker
• Magnetic stir plate with rod
• Glass test tubes
• Centrifuge
• Plastic disposable pipettes
• Cotton balls
• Plastic funnel
• Thermo Scientific spectronic 20D+ spectrophotometer
• Distilled water
• Plastic cuvettes

### Methods

Step 1 (Carbonization)

First, bamboo sticks were ground into shavings, and 6 grams of shavings were collected and added to a 15.24 × 7.62 × 10.16 cm metal container. It is important not to use aluminum in any of these procedures as the melting point is very close to the temperatures needed for activation. A metal lid with 6 small evenly-spaced 1.5 mm holes was pressed onto the container to seal and cover it. The sealed container was placed onto a propane burner and combusted. Smoke released from the perforations of the lid was monitored; when no more smoke was released, the container was removed from the fire. Immediately after the fire was extinguished, the carbonized bamboo was transferred into a mortar and ground by a pestle. Each sample was vigorously ground for 2–3 minutes.

Step 2 (Activation)

Ground, carbonized bamboo was placed in a small metal box. This box was placed back onto the propane burner with the lid open to allow exposure to oxygen for 6 minutes; a stainless-steel laboratory spatula was used to gently stir the charcoal during activation. An infrared thermometer gun was pointed to the surface of the charcoal to monitor its temperature every 10–15 seconds; the fuel knob was adjusted accordingly to keep this temperature between 450 and 550 °C. At this temperature the charcoal will glow a reddish orange lavalike color. If the temperature goes above 600 °C the activation does not work as well. Once 6 minutes had passed, the lid to the small metal container was quickly closed and the burner was turned off. Once the container was cool, the activated charcoal was ready for testing.

### Method validation

**Fig. 1:**
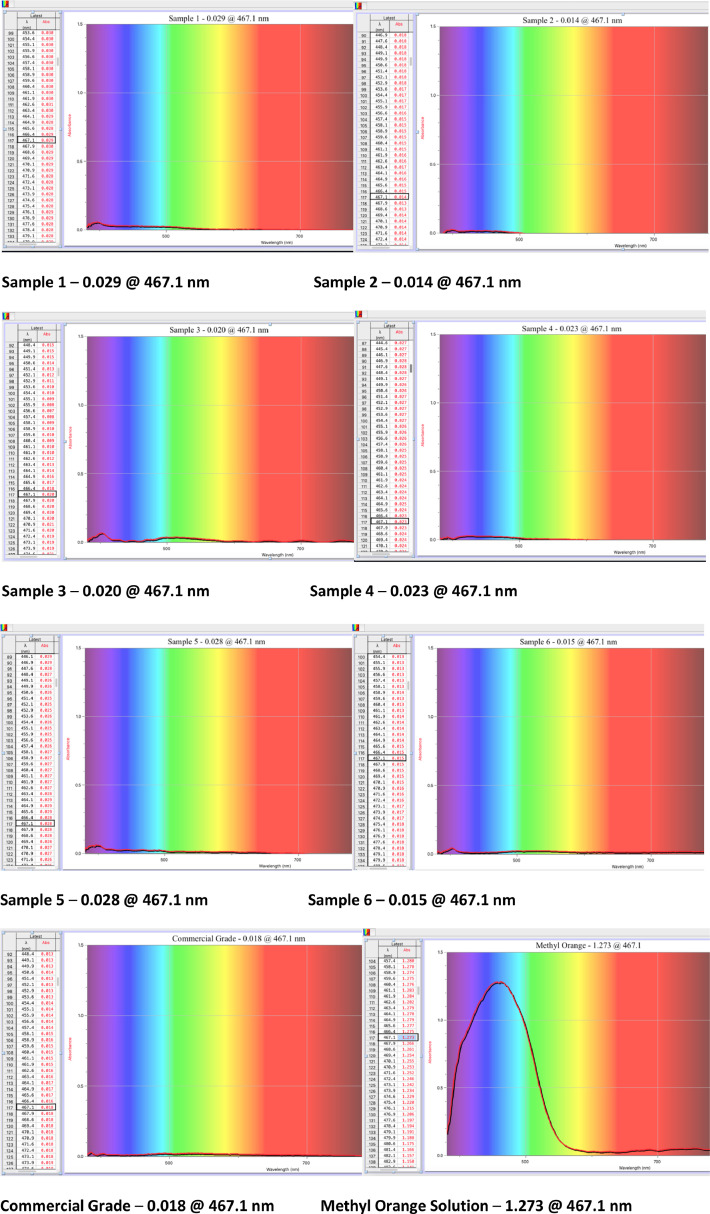
Spectrophotometric analysis of experimental samples and commercial grade.

**Fig. 2:**
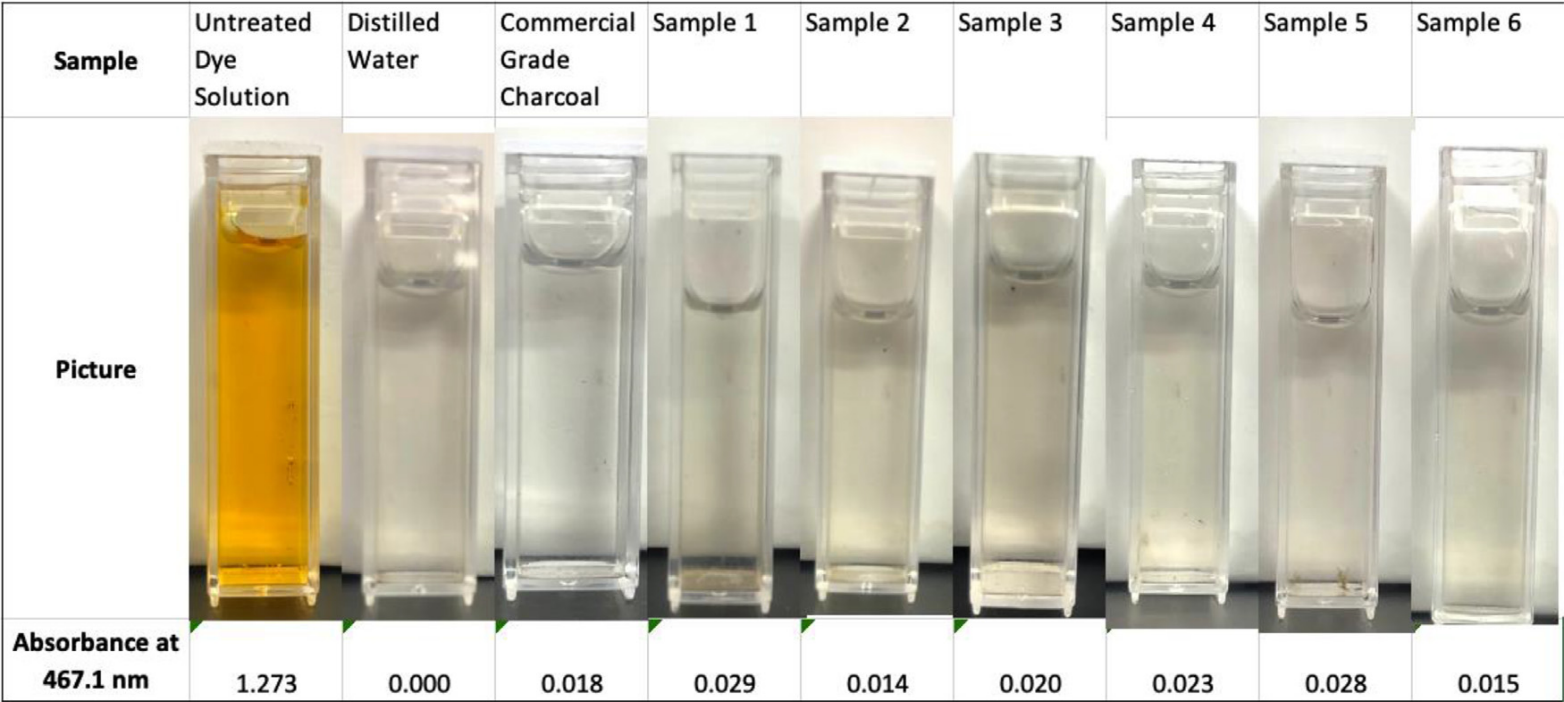
Visual inspection of adsorptive capacity vs untreated dye solution and distilled water.

Once cooled, 0.20 g of activated charcoal was taken from the small metal container and added to 25 mL of stock methyl orange solution in a beaker; methyl orange solution was made by mixing 0.025 g of methyl orange indicator powder to 1 liter of distilled water. This solution was stirred for one hour using a magnetic stir rod and stir plate. After the mixture of charcoal and methyl orange solution was stirred for one hour, the mixture was centrifuged twice for one-hour periods to remove the charcoal. After the first centrifugation, the resulting dye solution was decanted by a pipette into another test tube. After the second centrifugation, the decanted liquid was then filtered through cotton to remove any residual charcoal. The filtered solution was added to plastic cuvettes for spectrophotometric analysis. The resultant charcoal-treated-fluid of each sample was read by a spectrophotometer at 467.1 nm to obtain the respective absorbance value as seen in Fig. 1. Photos of each charcoal-treated-fluid were taken and are shown in Fig. 2. To ascertain how well this activation process worked, commercial grade charcoal was collected and tested by the same protocol above.

Six batches of bamboo shavings were carbonized and activated using the above methods. Following activation, spectrophotometric analysis was performed using a Thermo Scientific spectronic 20D+ spectrophotometer set to 467.1 nm. The stock methyl orange solution was tested as a negative control; distilled water was measured as a positive control (not shown, but had the expected absorbance of 0.000), and a 0.20 g sample of commercial grade charcoal was tested using the above stated testing method as a positive control. The six samples from bamboo shavings activated using the method described in this paper were tested as well. The results from the spectrophotometric analysis can be seen below in Fig. 1 and photos for visual analysis can be seen in Fig. 2. The absorbance of the solution that resulted after testing the adsorption of the six charcoal samples had a mean of 0.0215 ± 0.00237 (SEM) compared to an absorbance of 0.018 for commercial grade charcoal.

## CRediT authorship contribution statement

**Stephen Nelson:** Methodology, Validation, Investigation, Writing – original draft, Visualization. **Connor Hare:** Methodology, Investigation, Validation. **Atieno Mpyisi:** Investigation, Validation. **Chen Wei Yew:** Methodology, Investigation, Writing – original draft. **Kendra LeBrun:** Conceptualization, Methodology, Investigation. **Christina R. Harris:** Resources, Supervision, Writing – review & editing. **Berquin D. Feese:** Supervision, Writing – review & editing. **Ronald Celestine:** Supervision, Writing – review & editing. **Masato Kinjo:** Supervision, Resources, Conceptualization, Validation, Writing – review & editing. **John S. Peacock:** Supervision, Resources, Conceptualization, Validation, Writing – review & editing.

## Declaration of Competing Interest

The authors declare that they have no known competing financial interests or personal relationships that could have appeared to influence the work reported in this paper.

## Data Availability

Data will be made available on request.

## Appendix Supplementary materials

Image, application 1

