# Supplementary material for: A simplified methodology for the activation of organic charcoal
Source: MethodsX. 2023 Jun 13;11:102241. doi: 10.1016/j.mex.2023.102241 (PMC10293709; doi:10.1016/j.mex.2023.102241)
Supplement: Supplementary file 1 [file mmc1.docx]

**Supplementary material *and/or* additional information [OPTIONAL]**

It has been proposed that the greatest failure of the 20th century is the perpetuation of the consumption of water which is contaminated and unsafe for consumption. This has been contributing to a high rate of mortality among young children which could be prevented by access to safe and sanitized water (Gleick, 2002). The most important public health problem related to contaminated water is diarrhea. It has been estimated that yearly there are 4 billion cases of diarrhea resulting in 2.2 million deaths each year, mostly among children under the age of five (Lakshminarayanan & Jayalakshmy, 2015). The sources of contaminated water intended for consumption include spring water, surface water, and unprotected wells. The outbreaks of diseases such as Ebola and cholera are often not effectively contained as a result of the lack of an access to safe and sanitized water (WHO, 2015 Update and MDG Assessment, 2015). We propose that the results presented herein could help provide a partial solution to some of the challenges mentioned above. Activated charcoal can partially remove water contaminants such as suspended solids, chemical impurities, harmful and carcinogenic dyes, bacteria, bacterial spores, bacterial toxins, viruses, herbicides, pesticides and fertilizers residues: water contaminants that can cause blindness, poisoning, paralysis, sterility, flu-like symptoms, skin rashes, blurred vision, mild headaches and death. Thus, the risk of these health problems can be reduced by using activated charcoal to help eliminate some water contaminants making water safer (Cobb, Warms, Maurer, & Chiesa, 2012; Iqbal & Ashiq, 2007; Naka et al., 2001; Williams, 2000). Activated charcoal has been utilized as a treatment option for diarrhea and it can adsorb toxins in the intestinal tract of humans (Ilomuanya, Ifudu, & Uboh, 2011).

Current research demonstrates that activated charcoal can be synthesized in a laboratory using concentrations of strong acids such as sulfuric and phosphoric acid, strong base such as sodium hydroxide, and distilled water (Mkungunugwa et al., 2021). However, here we present an easier alternative for charcoal activation that is cost-effective using only simple household materials and organic matter. Our data indicates that this technique for activating charcoal consistently produces charcoal with an adsorptive capacity near to that of commercial-grade charcoal as measured by spectrophotometry and visual inspection. Spectrophotometry is often used in procedures that test the adsorptive capacity of activated charcoal (Zhang et al., 2010). Using this method, it is relatively easy to produce activated charcoal in an emergency situation or in a third world country with basic equipment. Therefore, this paper presents a partial solution to those that are challenged to live on a hazardous and contaminated water supply.

Work began with exploration of using sodium chloride for activation, and although we had some success, replicability of results and desalination of the charcoal proved challenging. Therefore, we began the process of exploring alternative methods of activation. Various other organic materials were experimented with and successfully used, but we did find that harder substances tended to do better (e.g. oak did better than pine.) Also, activation time was tested ranging from 3-8 minutes to find that 6 minutes was the ideal time for activation. Although this protocol ground the bamboo prior to activation preliminary data did not suggest that grinding made a substantial difference in activation, but was used primarily to speed up the process of carbonization.

**References**

Cobb, A., Warms, M., Maurer, E. P., & Chiesa, S. (2012). Low-Tech Coconut Shell Activated Charcoal Production. *International Journal for Service Learning in Engineering, Humanitarian Engineering and Social Entrepreneurship*, *7*(1), 93–104. <https://doi.org/10.24908/ijsle.v7i1.4244>

Gleick, P. H. (2002). Dirty-water: estimated deaths from water-related diseases 2000-2020 (pp. 1-12). Oakland: Pacific Institute for studies in Development, environment, and security.

Ilomuanya, M. O., Ifudu, N. D., & Uboh, C. (2011). The use of metronidazole and activated charcoal in the treatment of diarrhea caused by Escherichia coli 0157: H7 in an in vitro pharmacodynamic model. African Journal of Pharmacy and Pharmacology, 5(10), 1292-1296.

Iqbal, M. J., & Ashiq, M. N. (2007). Adsorption of dyes from aqueous solutions on activated charcoal. *Journal of Hazardous Materials*, *139*(1), 57–66. <https://doi.org/10.1016/j.jhazmat.2006.06.007>

Lakshminarayanan, S., & Jayalakshmy, R. (2015). Diarrheal diseases among children in India: Current scenario and future perspectives. *Journal of natural science, biology, and medicine*, 6(1), 24–28. <https://doi.org/10.4103/0976-9668.149073>

Mkungunugwa, T., Manhokwe, S., Chawafambira, A., & Shumba, M. (2021). Synthesis and characterisation of activated carbon obtained from Marula (Sclerocarya Birrea) nutshell. *Journal of Chemistry*, *2021*, 1–9. <https://doi.org/10.1155/2021/5552224>

Naka, K., Watarai, S., Tana, Inoue, K., Kodama, Y., Oguma, K., Yasuda, T., & Kodama, H. (2001). Adsorption Effect of Activated Charcoal on Enterohemorrhagic Escherichia coli. Journal of Veterinary Medical Science, 63(3), 281–285. <https://doi.org/10.1292/jvms.63.281>

Williams, C. (2000). Clinisorb activated charcoal dressing for odour control. British Journal of Nursing, 9(15), 1016-1019.

World Health Organization. (2015). Progress on sanitation and drinking water–2015 update and MDG assessment.

World Health Organization (WHO, & UNICEF). (2000). Global water supply and sanitation assessment 2000 report. World Health Organization (WHO).

Zhang, Z., Zhang, Z., Fernández, Y., Menéndez, J. A., Niu, H., Peng, J., Zhang, L., & Guo, S. (2010). Adsorption isotherms and kinetics of methylene blue on a low-cost adsorbent recovered from a spent catalyst of vinyl acetate synthesis. *Applied Surface Science*, *256*(8), 2569–2576. <https://doi.org/10.1016/j.apsusc.2009.10>.
